# Microbial diversity and component variation in Xiaguan Tuo Tea during pile fermentation

**DOI:** 10.1371/journal.pone.0190318

**Published:** 2018-02-20

**Authors:** Haizhou Li, Min Li, Xinrui Yang, Xin Gui, Guofeng Chen, Jiuyun Chu, Xingwang He, Weitao Wang, Feng Han, Ping Li

**Affiliations:** 1 Research Center for Translational Medicine at Shanghai East Hospital, School of Life Sciences and Technology, Tongji University, Shanghai, China; 2 Yunnan Xiaguan Tuo Tea (Group) Co., Ltd, Dali, Yunnan, China; 3 College of Architecture and Urban Planning, Tongji University, Shanghai, China; Chinese Academy of Medical Sciences and Peking Union Medical College, CHINA

## Abstract

Xiaguan Tuo Tea is largely consumed by the Chinese, but there is little research into the microbial diversity and component changes during the fermentation of this tea. In this study, we first used fluorescence in situ hybridization (FISH), next-generation sequencing (NGS) and chemical analysis methods to determine the microbial abundance and diversity and the chemical composition during fermentation. The FISH results showed that the total number of microorganisms ranges from 2.3×10^2^ to 4.0×10^8^ cells per gram of sample during fermentation and is mainly dominated by fungi. In the early fermentation stages, molds are dominant (0.6×10^2^~2.8×10^6^ cells/g, 0~35 d). However, in the late stages of fermentation, yeasts are dominant (3.6×10^4^~9.6×10^6^ cells/g, 35~56 d). The bacteria have little effect during the fermentation of tea (10^2^~10^3^ cells/g, <1% of fungus values). Of these fungi, *A*. *niger* (*Aspergillus niger*) and *B*. *adeninivorans* (*Blastobotrys adeninivorans*) are identified as the two most common strains, based on Next-generation Sequencing (NGS) analysis. Peak diversity in tea was observed at day 35 of fermentation (Shannon–Weaver index: 1.195857), and lower diversity was observed on days 6 and 56 of fermentation (Shannon–Weaver index 0.860589 and 1.119106, respectively). During the microbial fermentation, compared to the unfermented tea, the tea polyphenol content decreased by 54%, and the caffeine content increased by 59%. Theanine and free amino acid contents were reduced during fermentation by 81.1 and 92.85%, respectively.

## Introduction

Xiaguan Tuo Tea is a fully fermented black tea and belong to Pu-erh tea.[1]. It is mainly produced in Dali City, Yunnan Province, and is made from *Camellia sinensis*[2, 3]. This tea is a bowl-shaped compressed mass of tea leaves and appears reddish, brownish red or gray in color. Usually, Xiaguan Tuo Tea is produced by using an empirical fermentation process that is also called “pile fermentation”[4–6]. This fermentation process involves complex biological transformations by diverse microorganisms[1, 7, 8]. In general, three classic fermentation stages are required to produce Xiaguan Tuo Tea. First, fresh tea leaves are collected, dried, and piled into windrows. Second, the proper temperature and humidity are provided for 56 days to allow leaf oxidation when the piles are rolled over every 7 days. Finally, the end-product is dried and stored[9, 10].

Microorganisms play a key role in the fermentation process, both altering quality and transforming tea components. The researchers first isolated *Aspergillus*, *Trichoderma*, *Rhizopus*, and yeast from the black tea fermentation process[2, 3, 11] and found that *Aspergillus* or yeast is the dominant strain in the black tea fermentation[12]. *A*. *niger*, *A*. *glaucus*, *A*. *terreus*, *A*. *japonicus* var., *A*. *aureolatus*, *A*. *foetidus*, *A*. *candidus*, *A*. *egyptiacus*, *A*. *penicillioides* and *A*. *oryzae* were then identified in black tea fermentation[13]. Lyu[14] reported the diversity of yeast in Yunnan Pu'er tea microbes and found that *C*. *parapsilosis*, *S*. *kluyveri*, *C*. *famata*, *C*. *affeifera* and *C*. *laurentii* participated in the fermentation of this tea. At the same time, Gong et al. [9] isolated basidiomycetes from Pu’er tea. Ade[13] first used the PCR-DGGE method to investigate microbial diversity in tea fermentation and found *B*. *adeninivorans* during the fermentation of Pu'er tea. In addition, Tian et al[2] found that the yeasts *Aspergillus* and *Penicillium* were the main fungi involved in the fermentation of Pu’er tea.

During fermentation, the metabolism and reproduction of microbes dramatically change the chemical constitution of Xiaguan Tuo tea. The main components of tea include tea polyphenols, caffeine, proteins, amino acids, and carbohydrates[10, 15, 16]. They undergo secondary oxidization, condensation, decomposition and other processes caused by microbes growing in tea fermentation, thus making a unique type of Xiaguan Tuo Tea[17]. Until now, most reports have focused on Lincang and Puer city, Yunnan Province, China.

In this paper, tea samples were collected from the Dali tea factory in Yunnan Province. Culture-dependent, fluorescence in situ hybridization (FISH) and next-generation sequencing (NGS) approaches were used to systematically investigate the diversity and dynamics of microorganisms. We also monitored the temperature, water content, and pH under industrial fermentation conditions, in addition to analyzing the tea’s polyphenol, caffeine, total amino acid and theanine content on the lab scale.

## Materials and methods

### Tea sample collection

Xiaguan Tuo Tea was made from the leaves of the plant *Camellia sinensis* and obtained from Xiaguan, Yunnan, China (25°34’55.88”N, 100°12’41.88”E). The tea was produced by Yunnan Xiaguan Tuo Tea (Group) Co., Ltd in April 2015. We collected unfermented tea, first turn tea (6 d), second turn tea (11 d), third turn tea (19 d), fourth turn tea (27 d), fifth turn tea (34 d), sixth turn tea (40 d), seventh turn tea (47 d), and fermentation termination tea (56 d) samples.

### Fermentation process characterization

At the beginning of Xiaguan Tuo tea fermentation, water was scattered on the leaves until it reached approximately 30–35%. The wet tea was placed into a fermentation room. During tea fermentation, the tea was turned over seven times to control the fermentation temperature. When leaves were turned over, the temperature was measured in the core of the tea pile. The water content in the tea was calculated after drying the tea at 105°C for 1 hour. Then, 1 g of tea was suspended in 10 mL of deionized water by using a homogenizer at 150 rpm for 10 min, and the pH of the water was measured. Each sample was analyzed in triplicate, and the values were expressed as the mean (n = 3). When leaves were turned over, tea samples were removed for analysis of microorganisms and chemical components.

### Cultivation conditions of microorganisms in the Xiaguan Tuo Tea

First, 1 g of fermented tea was placed into 10 mL of sterile water. Then, the flask was shaken several times to break up microorganisms that had adhered to the tea. Then, 100 μL of water was transferred into 900 μL of sterile water. The procedure was repeated five times, and each time resulted in a ten-fold dilution of the previous tube. Finally, 100 μL of water from each dilution was transferred to plates of different culture media. The bacterial culture medium contained 5 g of peptone, 1.5 g of beef extract and 15 g of agar in 1000 mL of water; fungal culture medium contained 10 g of glucose, 15 g of agar, and 100 g of peeled potato in 1000 mL of water; yeast culture medium contained 10 g of glucose, 5 g of peptone, 2.5 g of yeast extract and 10 g of agar in 1000 mL of water. Ampicillin was added to the fungal and yeast media (10μg/mL). The incubation temperature and period were 30°C and 7 d, respectively. Each sample was done in triplicate, and the values were expressed as the mean (n = 3).

### Isolation of total DNA

The isolation of DNA from bacteria, fungi, and yeasts from tea samples was done using the Fast DNA SPIN Kit for Soil (MP Biomedical, USA), finally eluting in 50 μL of MQ water. The DNA was then stored at −20°C.

### Next-generation sequencing and sequence analysis

We amplified the ITS region using the Miseq-ITS primers ITS1FI2 and ITS2R. ITS1FI2: 5’-CCCTACACGACGCTCTTCCGATCTN(barcode)CTTGGTCATTTAGAGGAAGTAA, ITS2R: 5’-GTGACTGGAGTTCCTTGGCACCCGAGAATTCCAGCTGCGTTCTTCATCGATGC-3’. ITS1FI2 primer overlaps in six positions with ITS1F, but is located closer to the end of the 18S. Fragments in the size range of ITS1FI2 and ITS2R can be readily sequenced on the Illumina MiSeq platform. The Illumina platform provides sequencing at greater depth for a considerably lower price compared to 454 pyrosequencing, and this promises a deeper characterization of fungal communities. The barcode sequences are added to each sample so they can be distinguished and sorted during data analysis. PCRs were set up to run at 3 min at 95°C, followed by 5 cycles of 20 s at 95°C, 30 s at 65°C, 20 cycles of 20 s at 94°C, 20 s at 55°C, and 30 s at 72°C. A final elongation was done at 72°C for 5 min. Then, we used combinatorial primer labeling to identify samples after the first PCR. The second PCR conditions were 30 s at 95°C, followed by 5 cycles of 15 s at 95°C, 15 s at 55°C, and 30 s at 72°C. A final elongation was done at 72°C for 5 min. Amplifications were carried out in a total volume of 50 μL, using 20 ng of DNA, Taq polymerase (Thermo, USA), 100 mM KCl, 500 μM each dNTP, 3 mM MgCl_2_, 20 mM Tris-HCl (pH 8.3), and 0.4 μM each primer and PCR-enhancing substances. Purification was done with an Agencourt AMPure XP system (Beckman, USA). We normalized PCR products after quantifying them with a Qubit 2.0 Fluorometer(Invitrogen). Paired-end sequencing (2×150 bp) was carried out on an Illumina MiSeq sequencer at the Sangon Genome Center (Shanghai, China) using NGS. We assembled paired-end reads using PEAR[18]. The quality of the reads was checked by using PRINSEQ[19]. Chimera detection was performed with the USEARCH[20]. OTUs were picked at the 97% sequence identity level by USEARCH. One sequence from each OTU was selected to be representative, and the closest reference sequences (GenBank: http://www.ncbi.nlm.nih.gov & RDP) were pooled and aligned using CLUSTAL X[21]. Phylogenetic analysis was performed using the distance-based maximum likelihood method with MEGA 7.0[22]. Bootstrap analysis was performed using 1000 replications. The Shannon-Weaver and Chao1 diversity indices were calculated using MOTHUR[23]. Rarefaction curves were calculated using MOTHUR. Sequence data are publicly available via the NCBI Sequence Read Archive database (SRP091015).

### Microorganism counts in fermentation using FISH

All samples for FISH were collected at different time of tea fermentation and stored on dry ice during transportation. For detection, 0.5 g of tea sample was used. Then, 320 μL of 25%(w/v) particle free paraformaldehyde solution (4% final concentration) was added, flled up with 1× PBS, mixed up completely, and the suspension was stored at 4 °C for 24 h. The fixed samples were washed twice with 1× PBS, centrifuged at 10,000×g for 5 min at 4 °C after each washing, and stored in PBS/ethanol (1:1) at −20 °C for further processing. Then, 100 μL of the fixed sample was diluted with 900 μL of PBS/ethanol, and the mixture was dispersed by ultrasound with an ultrasonic probe at minimum power for 10 s using 1-s sonication pulses. Then, 20 μL of the sample was diluted in 10 mL of MQ water. This suspension was filtered through polycarbonate filters (0.2 mm pores, 25 mm in diameter). If the signal intensity is low, the tea sample dilution rate was reduced accordingly. After filtration, the filters were dipped in 0.1% low-melting point agarose and dried in an incubator at 46°C. The cell walls were permeabilized by addition of proteinase K solution (15 μg/mL, Roche) and were then subsequently incubated in 3% H_2_O_2_ to inactivate endogenous peroxidases. Air-dried filters and cut filter sections were used for hybridization. Filter sections were placed in a 1.5-mL tube and mixed with 300 μL of hybridization buffer (10% (w/v) dextran sulfate, 2% (w/v) blocking reagent (Roche, Germany), 20 mM Tris–HCl [pH 8.0], 0.1% (w/v) sodium dodecyl sulfate, 0.9 M NaCl, and 55% (v/v) formamide) and 1 μL of probe working solution (final concentration 0.028 μM). The probes and their sequences are shown in Table 1. The nonsense probe NONEUB was used as a control. After hybridization at 46°C for at least 90 min on a rotor, the filters were transferred to prewarmed washing buffer (20 mM Tris-HCl [pH 8.0], 5 mM EDTA [pH 8.0], and 3 mM NaCl, 0.01% (w/v) SDS) and incubated for 15 min at 48°C; the samples were then mixed with 1000 μL of amplification buffer (1×PBS [pH 7.4], 0.1% (w/v) blocking reagent, and 0.0015% H_2_O_2_,) and 1 μL of Alexa488 Tyramide (molecular probes, Life Technologies^TM^). Then, the filter sections were incubated in amplification buffer at 46°C for at least 20 min in the dark. Afterwards, the filters were stained with DAPI and mounted with 5 μL droplets of antifade reagent (Molecular Probes, Life Technologies^TM^). Cell counting was performed on 10 randomly selected micrographs that were taken with 20× objectives (150,415 μm^2^), and the results were extrapolated to 1 g of tea. Automated counting was performed on micrographs that exhibited a high contrast between stained cells and background fluorescence with the image analysis software ImageJ [24–28].

**Table 1:** Oligonucleotide probes used in FISH experiments.

Probe Name	Target	Sequence (5’–3’)
EUB338 (I–III)	Bacteria	Mixture of the probes EUB338, EUB338 II, and EUB338 III
EUB338 I	Most Bacteria	GCT GCC TCC CGT AGG AGT
EUB338 II	Planctomycetes	GCA GCC ACC CGT AGG TGT
EUB338 III	*Verrucomicrobium*	GCT GCC ACC CGT AGG TGT
Yeast	All yeasts	CTC TGG CTT CAC CCT ATT C
Fungi	most fungus	TCC GTA GGT GAA CCT GCG G
Control	Nonsense of microbes	ACT CCT ACG GGA GGC AGC

### Determination of tea polyphenol content

The total polyphenol content in Xiaguan Tuo Tea was measured by using the Folin–Ciocalteu method. The Folin regent was made by adding 5 g of phosphomolybdic acid, 25 g of sodium tungstate, and 12.5 mL of phosphoric acid to 180 mL of distilled water and boiling the solution for 2 h. Then, the volume was made up to 1 L with distilled water. Next, 1 g of tea was boiled in 100 mL of distilled water for 1 h, and the solution was filtered to remove residual solids. In total, 5 mL of the extract was mixed with the same volume of Folin regent and left for 3 min. Then, 5 mL of sodium carbonate was added, and the solution was left for 1 h. The reaction mixture was centrifuged at 3000 rpm for 5 min, and the absorbance at 700 nm of the supernatant was measured. Each sample was done in triplicate, and the values were expressed as the mean (n = 3)[13].

### Determination of caffeine content

In total, 1 g of tea was extracted with 100 mL of boiling water for 60 min. 25 mL of the above solution was mixed with equal volume of chloroform (Sinopharm Chemical Reagent Co., Ltd, China) to extract caffeine from the tea. Caffeine was subsequently extracted into chloroform from the solution by using a separatory funnel. Finally, the absorbance of the solution was measured by using a UV spectrophotometer at 276 nm against the corresponding reagent blank. Each sample was done in triplicate, and the values were expressed as the mean (n = 3).[29].

### Determination of free amino acid and theanine content

Free amino acid and theanine content was determined using an Agilent HPLC instrument (Agilent Technologies, USA). HPLC separation was carried out using a C18 analytical column (250 mm×4.6 mm, 5 μm, Agilent, USA) maintained at 30°C. Gradient elution was used to obtain adequate separation. The mobile phase consisted of solvents A (0.1 M NaAc:ACN 97:3, v/v, pH 6.5) and B (ACN:water 4:1, v/v). The flow rate was 2 mL/min. Absorbance at 254 nm was measured using a UV detector. The total run time was 35 min. The sample injection volume was 2.0 μL. The analytical data were processed using Agilent software. Each sample was done in triplicate, and the values were expressed as the mean (n = 3)[30].

## Results

### Microbial diversity by dilution plating method

**Fig 1:**
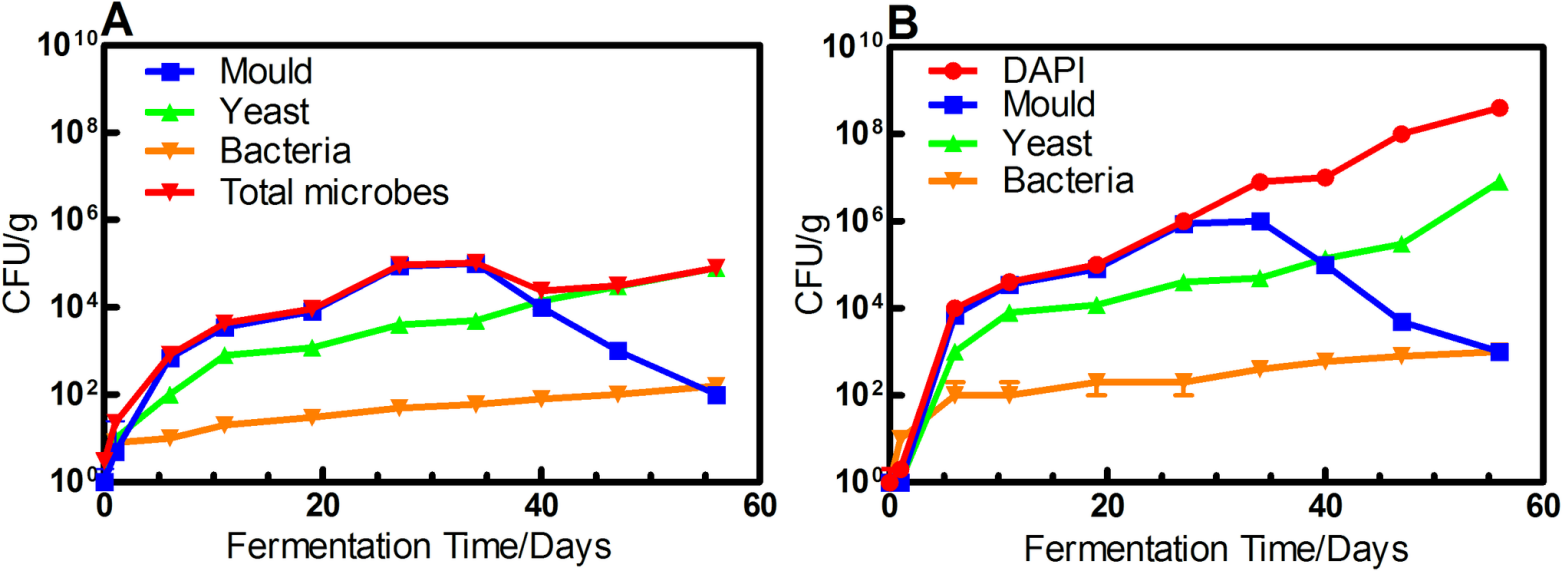
The number of microorganism during the fermentation of the pile of Xiaguan Tuo Tea. (A)analysis of the number of microorganisms by dilution plating method. (B)analysis of the number of microorganisms by FISH.

Microbe numbers and diversity in Xiaguan Tuo tea were determined using the dilution plating method. The data showed that the numbers of microorganisms increase from the black to the final product, ranging from 0.8×10^2^ to 8.6×10^5^ CFU/g (Fig 1A). The number of microbes in the early stages of the fermentation was small, but after the first turnover pile, the number of microbes began to increase, peaking in the last stage of fermentation. In the fermentation process, we found there were noticeable colonies of fungi (green, black, and white mold and yeast) by plate culture. Sequence analyses of 16S rRNA and 18S rRNA genes of colonies indicated that the black colony is a close relative of *Aspergillus* (DQ207726), the green colony is *Penicillium* (JH993675) or *Rhizopus* (CH476732), and the white colony is *Trichoderma* (NR134372). In the later stages of fermentation, fungi were isolated, and sequence analysis of their 18S rRNA genes revealed that they are affiliated with *B*. *adeninivorans* (KM409714) and *D*. *hansenii* (NC006043). At the 35th day of fermentation, the microbe numbers were the highest, and the microbial community and diversity were the most abundant. Furthermore, it was found that the distribution of different strains changed greatly during fermentation process. *A*. *niger* is the dominant strain in the early stage of fermentation. At 35 days, the *A*. *niger* content is at its maximum, reaching 8.4×10^4^CFU/g; it then gradually decreased to 1.2×10^2^CFU/g during late fermentation. *B*. *adeninivorans* and *D*. *hansenii* began to appear on the tea leaves at 35 days. The amount of *B*. *adeninivorans* increased gradually during the late stages of fermentation process and became the dominant strain (6.2 × 10^5^CFU/g).

### Microbial community dynamics by FISH

**Fig 2:**
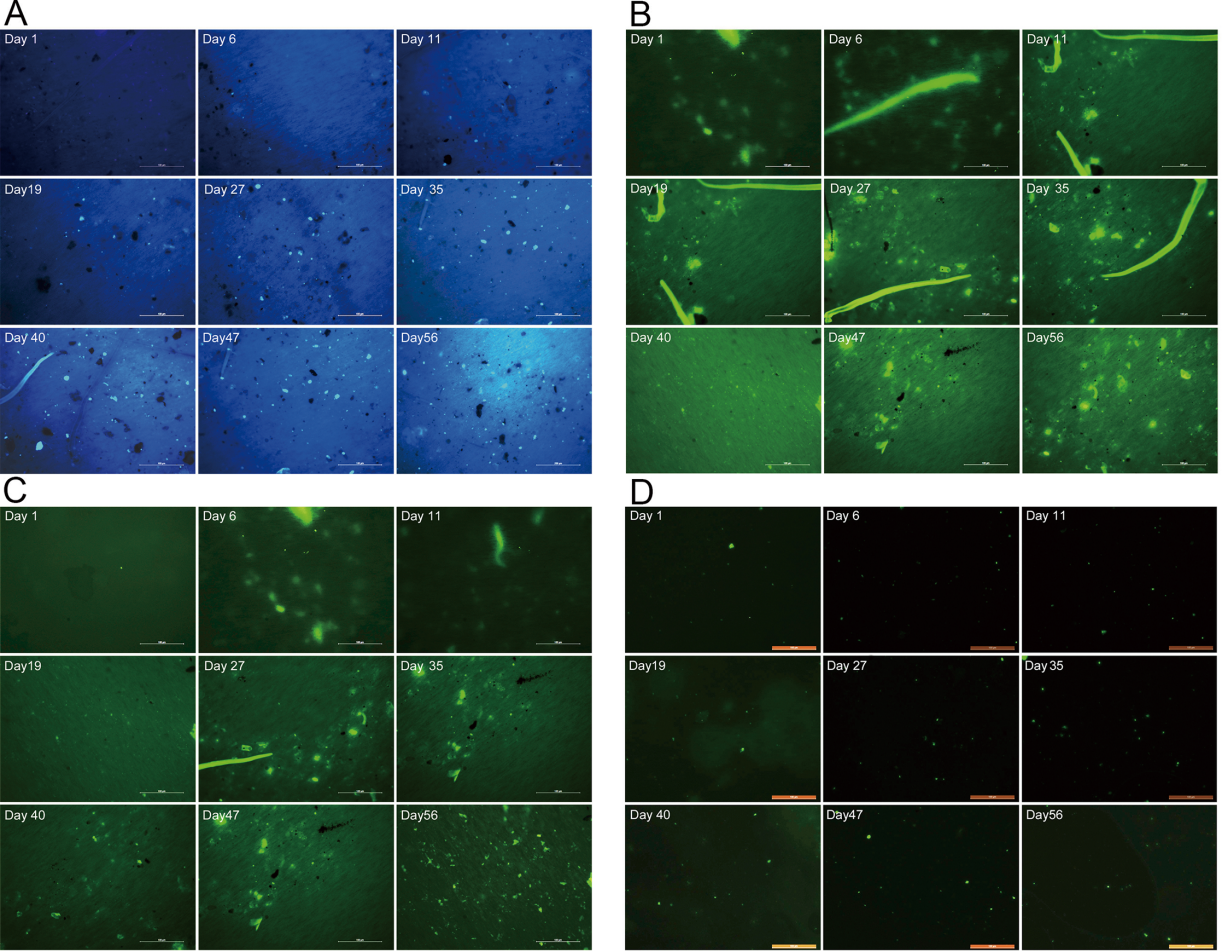
Photomicrographs of FISH stained samples. (A) DAPI stained. (B) Probe ITS targeted most of Fungi. (C) Probe Yeast targeted most of Yeast. (D) Probe EUB338 targeted most of Bacteria. Magnification = 200×, scale bar = 100 μm.

Furthermore, microbe populations could be accurately assessed by FISH. We used the FISH method to investigate the dynamics of microbes at nine different fermentation times (Fig 2, Table 1). These results indicated that during the fermentation of Xiaguan Tuo tea, the numbers of microorganisms greatly changed with the fermentation process. The total numbers of microorganisms was 2.3×10^2^cells/g at day 1 of fermentation, and the total numbers of microorganisms was 4.0×10^8^cells/g at day 56 of fermentation (Fig 1B). It was found that fungi always occupied a dominant position during the fermentation process, and bacteria were only a small fraction (<1%). At the same time, when the differences in microbe populations that were determined by the dilution plating method are compared with those obtained by the FISH method, the number of microbes determined by dilution plating is only 1% of the number of microbes by found by FISH. The reason for this difference is that some microorganisms could not be cultured by the culture-dependent method. Therefore, such culture-dependent methods barely delineate the true microbial diversity in the fermentation process of tea. Throughout the fermentation process, the number of molds increased during the initial fermentation and then decreased in the later stages of fermentation, and the results showed that the population of molds increased until around day 35 (Fig 1B). At day 1 of fermentation, the concentration of molds were 0.8×10^2^cells/g; it then increased slightly until one week had passed. By day 35 of fermentation, it had increased to 2.8×10^6^cells/g. Then, the number of molds decreased until the end of fermentation, when it reached 2.4×10^3^cell/g (Fig 1B).

**Fig 3:**
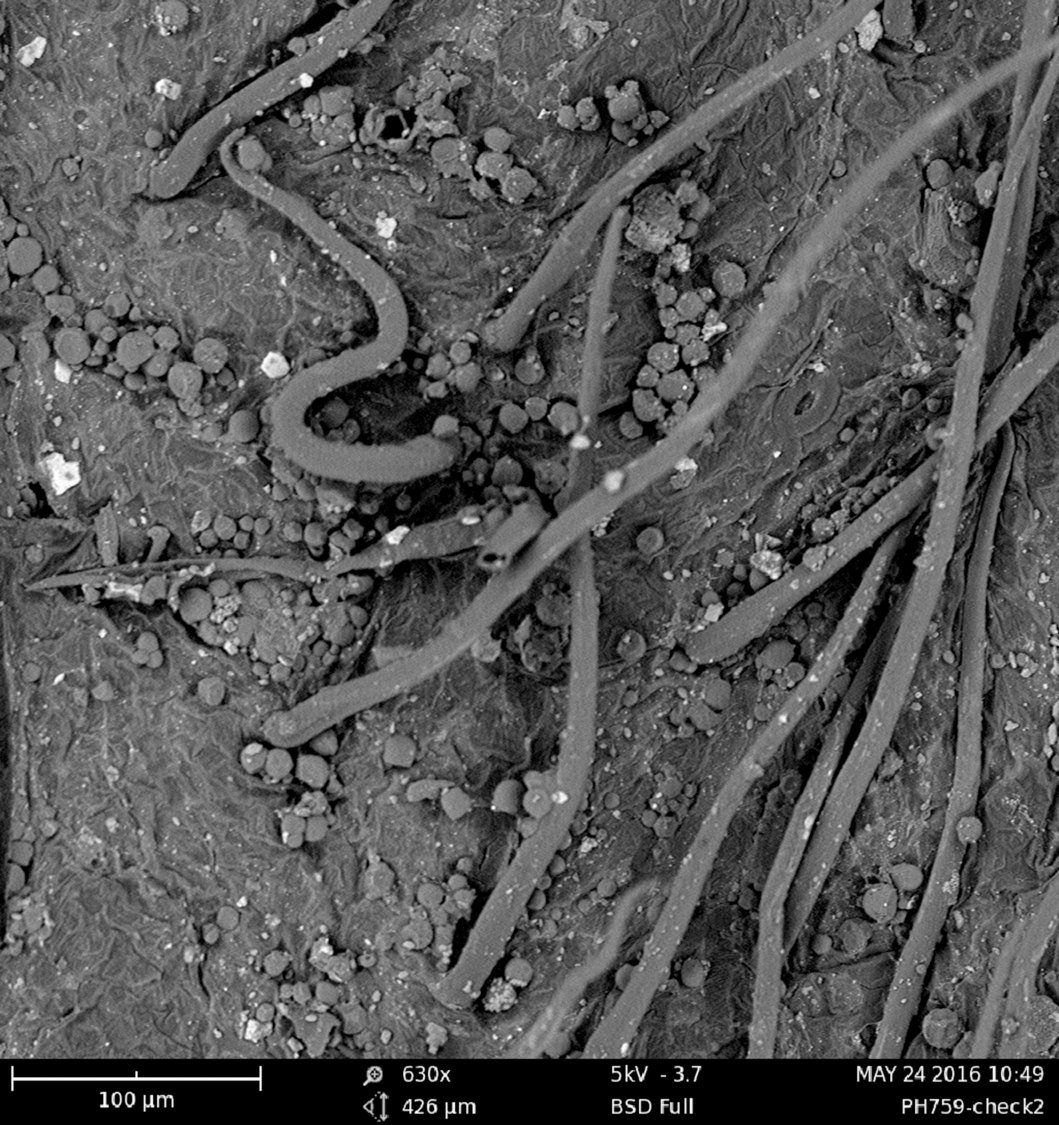
Photomicrograph of microbes in tea fermentation. Scale bar represents 100μm.

In the early stages of fermentation (0~35d), mold is the dominant microorganism; however, during the late stages of fermentation (35~56d), yeast is the dominant microorganism. While the population of yeast at days 0 to 6 was below the detectable level, 0.6×10^2^ cells yeast/g had appeared as early as day 6 of fermentation. From days 6 to 35, the number of yeast was lower than that of molds in tea, from 0.6×10^2^ to 3.6×10^4^ cells/g. The numbers of yeast then increased steadily until the end of fermentation, reaching 9.6×10^6^ cells/g. This pattern is consistent with the trend of the data from plate cultures. We also used SEM to observe the microbial morphology of microbes during the fermentation process (Fig 3).

### Fungus diversity by NGS

**Fig 4:**
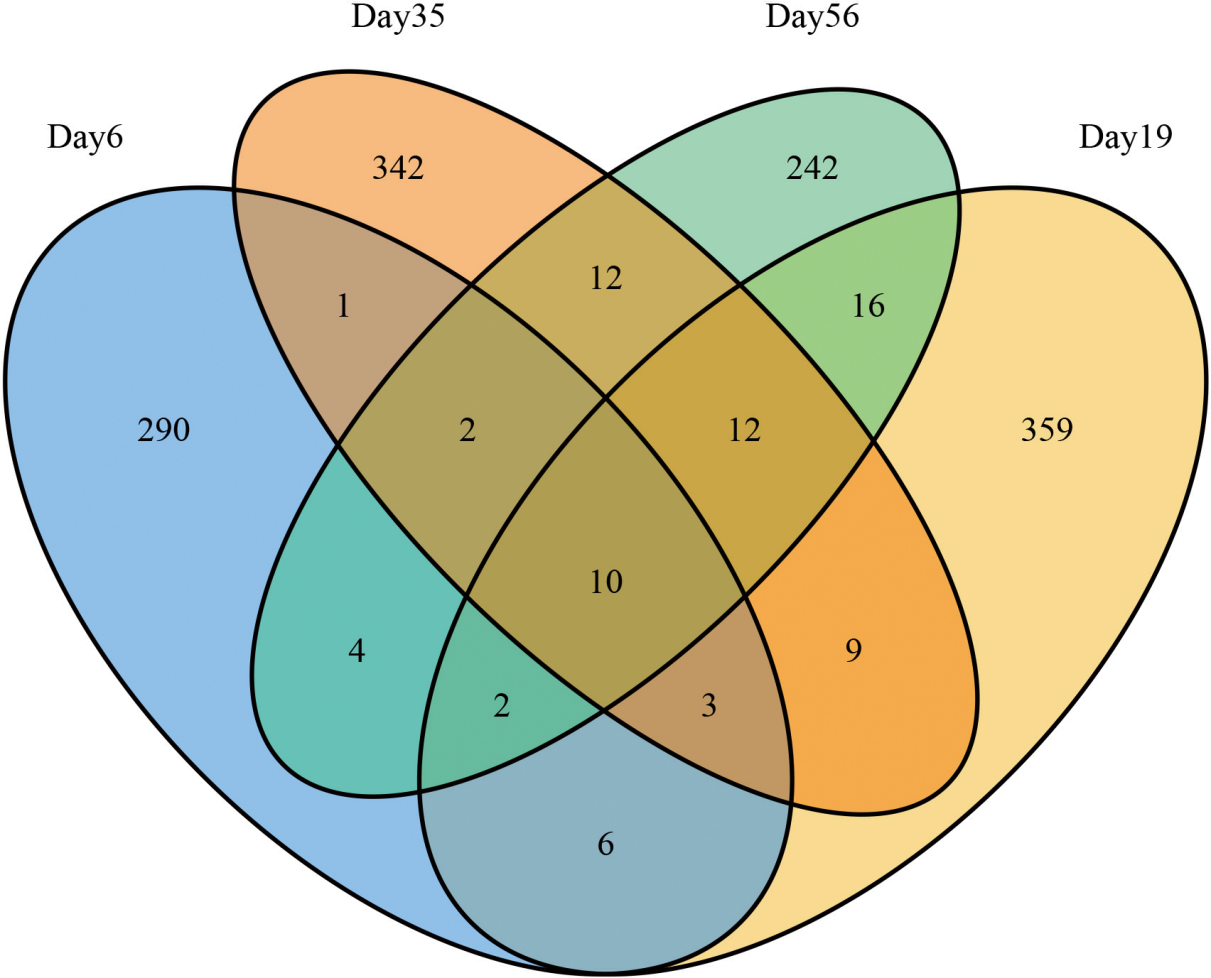
Distribution of OTUs (97% similarity) among the four different fermentation time.

**Fig 5:**
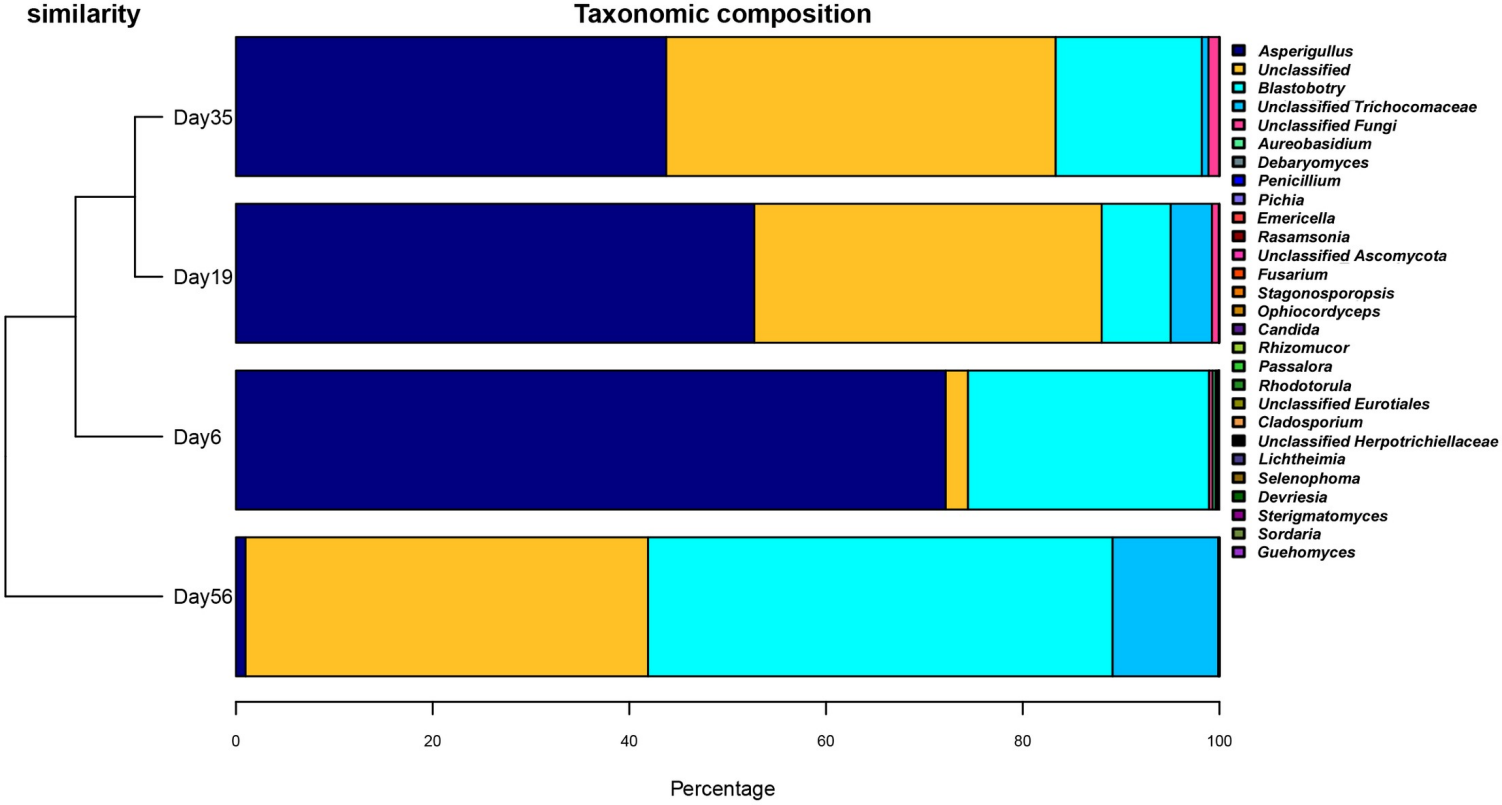
The microbial communities in different fermentation time.

**Fig 6:**
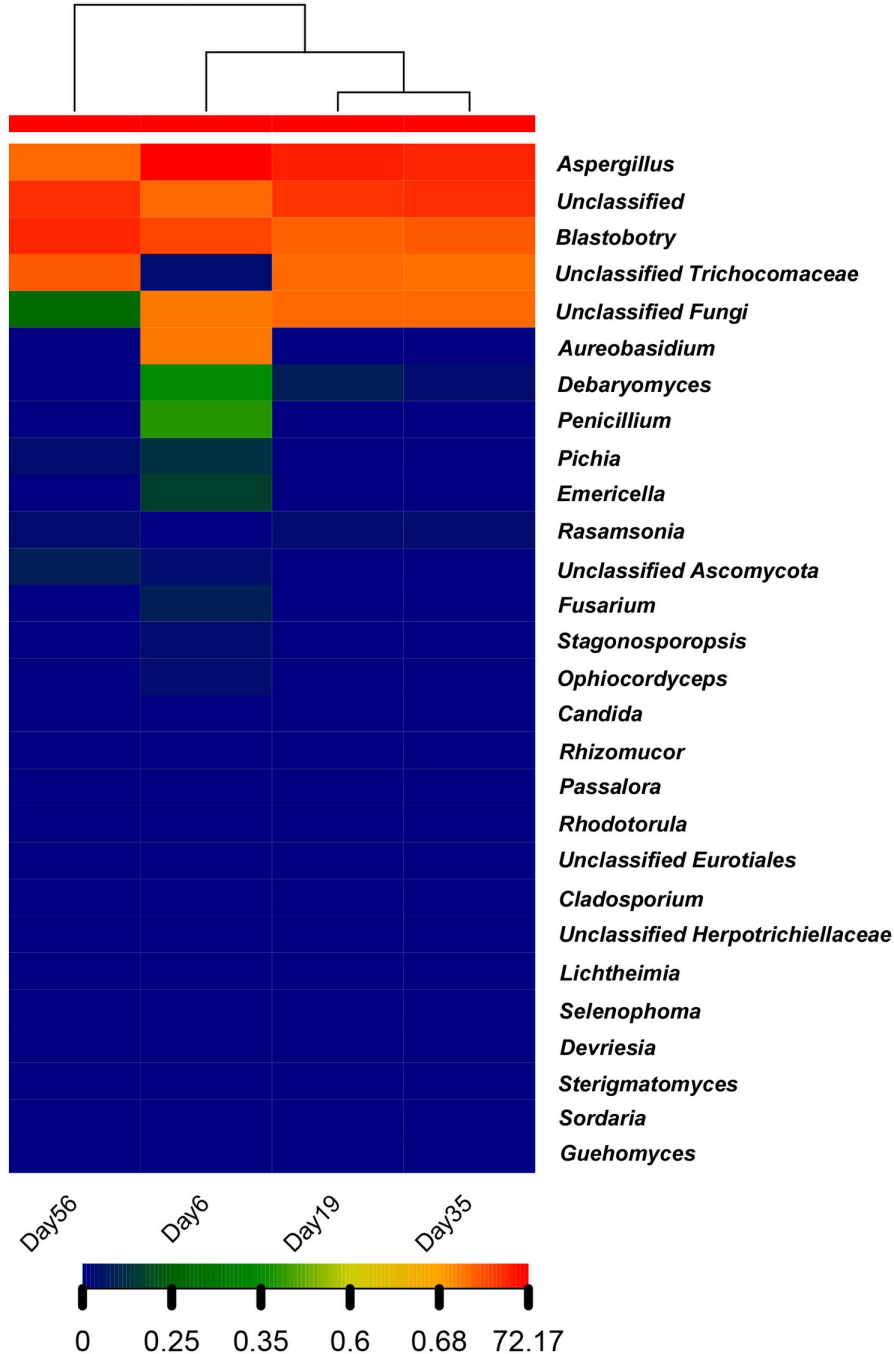
The heatmap of microbial communities in fermentation of tea.

Since the FISH results demonstrated that bacteria have little effect on pile fermentation, we only analyzed the fungus diversity in the fermentation process. A total of 222,029 ITS reads (Table 2) were obtained from the Miseq system (Illumina, USA). Quality checking resulted in 218,058 sequences, with an average length of 200 bp. DNA sequences were grouped in to respective OTUs at 97% sequence identity. In each tea sample, approximately 50,000 high-quality ITS sequences were obtained, and they were identified by comparing their sequences with those in the NCBI, SILVA and RDP RNA databases. From this approach, a range from 300~417 OTUs were detected in each sample (Table 2, Fig 4). OTUs representing 15 orders of fungi were detected. The phylogenetic distribution of the microbes found in Xiaguan Tuo Tea is shown in the tree in S1 Fig. At the phylum level, according to the dataset, the Ascomycota (152,697 reads) dominated both sequence sets, claiming >70% of the share during fermentation of Xiaguan Tuo Tea. In total, 20% of the reads are unclassified. The dominant orders among the in Ascomycota were Eurotiales and Saccharomycetales (46.51 and 23.41% of reads, respectively). A small proportion of Ascomycota were assigned to the Dothideales (0.072%), Hypocreales (0.007%), Pleosporales (0.0007%), Capnodiales (0.0004%), Mucorales (0.0002%), Sporidiobolales (0.0002%), Chaetothyriales (0.0002%), Agaricostilbales (0.0002%), Sordariales (0.0002%), and Cystofilobasidiales (0.0001%). Further taxonomic analysis of the Eurotiales class revealed that *Aspergillus* was the most abundant genus (Fig 5). The taxonomic analysis of yeast showed that *B*. *adeninivorans (88*.*8%)*, *Debaryomyces*(0.12%), *Pichia* (0.03%) were the three most abundant subgroups. A small proportion of OTUs were assigned to *Candida* (0.01%) and *Rhodotorula*(0.01%) (Fig 6).

**Table 2:** Diversity indices at the 97% OTU level of ITS gene fragments by NGS in each sample.

Sample	Seq num	OTU num	Shannon index	ACE index	Chao1 index
Day6	55010	318	0.860589	5577.712	2614.867
Day19	55688	417	1.184038	14604.855	4432.588
Day35	53007	391	1.195857	21686.316	7571.000
Day56	54353	300	1.119106	9673.376	2596.867

Globally, 28 fungus genera were identified. The other genera are those of unclassified fungi. The genera detected in the fermentation of Tuo tea included *Rasamsonia (emersonii)*, *Ophiocordyceps*, *Penicillium*, *Aureobasidium*, *Debaryomyces*, *Pichia*, *Emericella*, *Rasamsonia*, *Fusarium*, *Stagonosporopsis*, *Candida*, *Rhizomucor*, *Passalora*, *Rhodotorula*, unclassified Eurotiales, *Cladosporium*, unclassified Herpotrichiellaceae, *Selenophoma*, *Devriesia*, *Sterigmatomyces*, *Sordaria*, *Guehomyces*, *Chaetomium*, *Rhizopus*, *Alternaria*, *Emericella*, *Cordyceps*, *Cephaliophora*, and *Lichtheimia*. There are also many unknown fungi in the tea fermentation samples (S1 Fig).

### Characterization of the fermentation process

**Fig 7:**
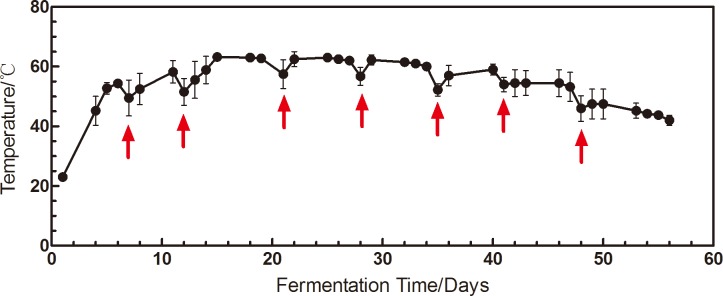
The temperature of the pile of tea leaves during the fermentation process for Xiaguan Tuo Tea. Values are expressed as mean±SD(n = 3). Red arrow indicate turnover of the pile of tea leaves to control the temperature.

**Fig 8:**
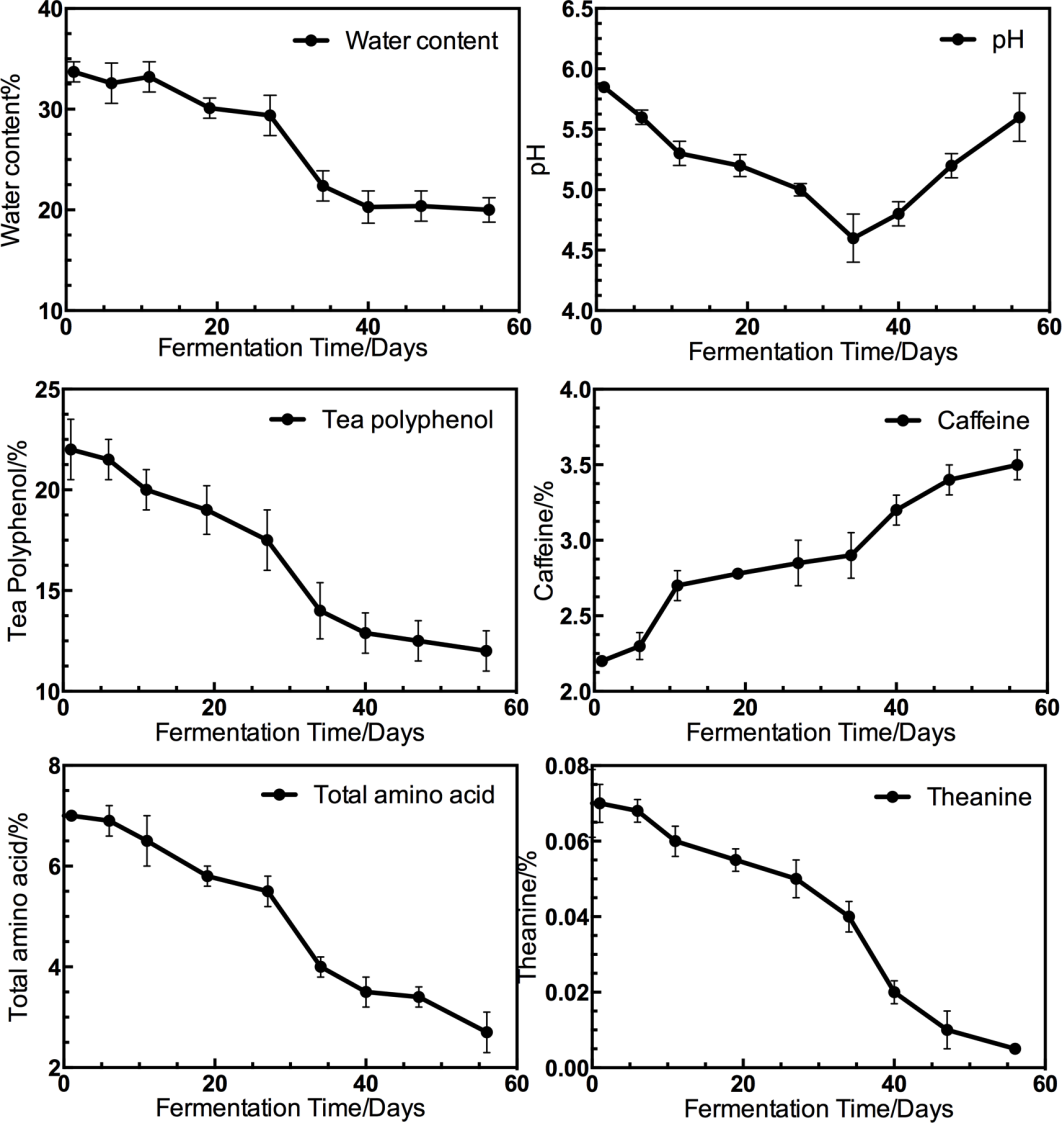
Water content, pH, polyphenol content, caffeine content, total free amino acid and theanine in the tea leaves during the fermentation process for Xiaguan Tuo Tea. Values are expressed as mean ± SD(n = 3).

The temperature, pH and water content of the piles were measured in the fermentation of Xiaguan Tuo Tea. The data showed that the temperature increased rapidly to 45°C at the beginning of fermentation and then stayed in the range from 55–65°C until day 35. The temperature then decreased to 39.5°C during the period from day 35 to 56 (Fig 7). At the beginning of the fermentation of the Xiaguan Tuo Tea, the water content was adjusted to 35%. Then, the water content decreased during fermentation, reaching 15% at the end of fermentation. The initial pH before fermentation was 5.85, but the pH value decreased to 4.5 at day 36 before increasing to 5.3 at the end of the fermentation. The water content remained below 35%, and the pH value was between 4.6 and 5.85 during tea fermentation (Fig 8).

### Tea polyphenol content

The concentrations of tea polyphenols were measured by using the Folin–Denis method, and the results are shown in Fig 8. The tea polyphenol content decreased from 21.9%, at the start to 12% by the end of 56 days of fermentation. The relative amounts of tea polyphenol content, compared to those of the unfermented tea, decreased by 54% during the 56 days of fermentation. Data show that tea polyphenol content decreases during the Xianguan Tuo Tea fermentation. The main reason is that tea polyphenols are enzymatically oxidized by microbes.

### Caffeine content, the total amino acid and theanine content

The amount of caffeine increased with fermentation time, and the initial total caffeine content was 2.2%, at the end of fermentation (day 56) the caffeine content was 3.5%. The relative amount of caffeine in the fermented tea, compared to the unfermented, increased by 59% during the 56 days of fermentations(Fig 8). Free amino acids are essential components in Xiaguan Tuo Tea. For instance, theanine is a very important contributor to the taste of Xiaguan Tuo Tea. Our data showed that the total free amino acid and theanine content was greater in raw tea than fermented tea (Fig 8). The initial free amino acid content was 7%, but the content started to decrease as fermentation progressed, finally reaching 2.7% (day 56). At the same time, the content of theanine dramatically decreased (p<0.05) during the fermentation of Xiaguan Tuo Tea. The initial theanine content was 0.07%, but the content decreased to 0.005% at the end of fermentation (day 56). The theanine contents decreased from its value in the raw tea by 92.85% after a 56-day fermentation. The transformation of total amino acids involves some complex reactions such as conversion by microbial enzymes and the Maillard reaction. We believe that these changes during the tea fermentation contribute to the color and flavor of tea. In addition, theanine accounted for approximately 11.6% of the total amino acids in Xiaguan Tuo Tea.

## Discussion

In our study, we used culture-dependent, fluorescence in situ hybridization (FISH), next-generation sequencing (NGS) and chemical analysis methods to comprehensively determine the microbial abundance and diversity and the chemical composition during the tea fermentation. We also investigated how microorganisms effect component transformations in tea. We found that the number of microorganisms continuously increased throughout the fermentation process. The number of microorganisms in the tea was measured using the dilution plating method and FISH, and the number of microorganisms determined by these techniques ranged from between 10^2^ and 10^5^/g of sample to between 10^2^ and 10^8^/g of sample, respectively (Fig 1). One finding was that the microbial populations determined by the plating method and by FISH differed. These results may be due to the presence of some microbes that could not be cultured. It is currently known that culturable microorganisms account for only 1% of the total amount of microbes in nature[31]. Meanwhile, the high-temperature, weakly acidic environment in the Xiaguan Tuo Tea fermentation process is difficult to completely simulate in the laboratory. Therefore, the culture-dependent method can only barely represent the true microbial diversity in the fermentation process of tea. We thus used the culture-independent method to investigate the number and diversity of microbes in the tea fermentation samples as it can accurately describe the microbe community structure during fermentation.

Furthermore, the fungus community structure was analyzed using the NGS method. A very obvious change in tea community diversity was apparent throughout the fermentation. At the early stages of fermentation (0~35d), molds accounted for the overwhelming majority of the microbial community (0.8×10^2^~2.8×10^6^ cells/g). However, in the late stages of fermentation (35~56d), yeasts accounted for the overwhelming majority of the microbial community (3.6×10^4^~9.6×10^6^cells/g). *A*. *niger* dominated from day 6 to day 35 of fermentation, and *B*. *adeninivorans* dominated from day 35 to day 56 of fermentation (Fig 5). This phenomenon was due to the rate of water dispersion by the leaves at the beginning of the fermentation. Humid environments are conducive to the growth of *A*. *niger*. The scattering of water on leaves is an important step in the fermentation. The water content of raw tea is 9~12%, so increasing the water content in the tea leaves is a necessary step for fermentation to occur. This humid environment favors the propagation of microorganisms. At the same time, it will help the microbes to produce a series of enzymes to catalyze chemical reactions. This step is very important, contributing to the quality of Xiaguan Tuo Tea. Therefore, in the factory, tea fermentation requires added water content (to a final level of 35%). When nutrition, temperature, humidity and other factors meet the growth needs of *A*. *niger*, this organism will decompose and intake sources of carbon and nitrogen. The metabolism of microbes results in a temperature rise. Thus, during the early stages of fermentation, *A*. *niger* accounted for the overwhelming majority of the microbial community.

Microbial metabolism releases heat, leading to increased temperatures; these warmer temperatures promote the growth of microbes. However, the higher temperature also inhibits the activity of microorganisms. Thus, during the tea fermentation process, turning the tea over is a key step in developing its quality. The manufactory maintains the temperature below 70°C by turning the tea pile over; in this manner, the tea in the fermentation can be fully decomposed by the microbial enzymes in the hot and humid environment. The arrows in Fig 7 show the turning over conditions. The temperature of the tea leaves is kept between 55 and 65°C throughout the fermentation process. This temperature can inhibit bacterial reproduction.

The water content is continuously reduced during the fermentation progress, resulting in deterioration of the growth conditions of *A*. *niger*. During the late stages of fermentation, the environment is unfavorable for *A*. *niger*, and yeasts begins to multiply. Yeasts depend on molds to decompose complex components, e.g., molds lyse complex carbohydrates such as lignin or cellulose into oligo- and mono-saccharides[32]. These components could support yeast growth[24]. Yeast thus dominates in the late stages of fermentation. As a result, a very obvious change in tea community diversity was apparent during the fermentation. The results were compared with those of Abe and the microbial trends in the fermentation of Yunnan Pu'er tea are similar to the trends in this study [13]. In the fermentation process, there are other fungi such as *Penicillium* and *Rhizopus*. *Penicillium* can secrete some antibacterial substances that can inhibit the growth of bacteria during tea fermentation. It can thus make the tea safer to drink.

During the middle stages of the fermentation (35d), the fungus community and diversity were the most abundant. The peak microorganism diversity in tea was observed at day 35 of fermentation (Shannon–Weaver index: 1.195857, Chao1: 7571.000), and less diversity was observed on days 6 and 56 (Shannon–Weaver index of 0.860589 and 1.119106, respectively). These results indicate that during the middle stages of fermentation, microbial metabolic activity is highest. This result is also consistent with the trend of the data from the plate culture method, which showed different kinds of fungi growing on the plates. During early stages of fermentation, molds breakdown complex compounds into small molecule carbon sources; for instance, complex carbohydrates such as lignin are converted into oligo- and mono-saccharides, which increases the diversity of available substrates [33, 34]. This range aids the survival of different kinds of microbes.

In addition, due to the variety of enzymes present, the metabolism and reproduction of microbes dramatically change the chemical composition of Xiaguan Tuo Tea. During microbial fermentation, compared to those of the unfermented tea, tea polyphenol content decreased by 54% and caffeine increased by 59%. Theanine and free amino acid contents were reduced during fermentation by 81.1 and 92.85%, respectively. Auto-oxidation or oxidation by associated fungi have been proposed to improve the taste and quality of tea[35]. For example, laccases play an important role in catalyzing the oxidation of tea polyphenols[36–38]. Laccases catalyze tea polyphenols into tea pigments and other compounds[39]. When tea polyphenols oxidize, they lead to the formation of theaflavins, bisflavanols, thearubigins, and theabrownin[39]. Tea pigments have been proven to have great beneficial effects for humans, including antiviral, antibacterial, anticancer and antioxidant activities[36–38]. XG Wang[40] also reported that caffeine in tea increased after the pile fermentation. It has been suggested that the changes in caffeine are due to the reproduction and growth of fungi and that caffeine can be produced by fungal enzymes[40, 41]. Free amino acids were consumed by microbe metabolism and the Maillard reaction. Both pathways change the total free amino acid content, but microbe metabolism is more important.

Previous studies have reported that *B*. *adeninivorans* has the gene for coding NADPH- and NADH-dependent monooxygenases and dioxygenases[42]. Moreover, it has been demonstrated that *B*. *adeninivorans* can grow at the expense of several benzene compounds. Based on these reports, we presume that *B*. *adeninivorans* can secrete some enzymes that oxidize tea polyphenols. Furthermore, *B*. *adeninivorans* secretes several extracellular enzymes, including cellobiases, xylosidase, proteases, phytase, and glucoamylase[43]. Therefore, *B*. *adeninivorans* has a significant effect on the transformation of the tea components. In summary, special fermentation processes facilitate the unique distribution of microbial diversity during different stages of fermentation, which leads to a significant change in biologically active components in Xiaguan Tuo Tea.

## Supporting information

S1 Fig Maximum likelihood phylogenetic tree. Maximum likelihood phylogenetic tree. Bootstrap (1,000 replicates) values of >50 are indicated at the nodes. The scale bar represents the estimated sequence divergence.
(TIF)
